# Retained gallbladder secondary to retrieval bag rupture during laparoscopic cholecystectomy—A case report

**DOI:** 10.1016/j.ijscr.2019.04.052

**Published:** 2019-05-09

**Authors:** Roy Huynh, Mark Magdy, Lucia Saliba, Ken Loi

**Affiliations:** a Faculty of Medicine, University of New South Wales, Sydney, Australia; b Department of Surgery, Fairfield Hospital, Sydney, Australia; c St George Private Hospital, Kogarah, New South Wales, Australia

**Keywords:** Laparoscopic cholecystectomy, Surgery complications, Retrieval bag rupture, Gallbladder remnant, Intra-abdominal abscess, Case report

## Abstract

**Introduction:**

Retrieval bags are used in laparoscopic cholecystectomies to reduce the risk of bile and gallstone spillage during removal of the gallbladder. Retrieval bag rupture is rare, and its complications have never been previously documented.

**Presentation of case:**

A 17-year-old female presented three months post-laparoscopic cholecystectomy with a tender periumbilical mass. Her operative report noted difficulty removing the retrieval bag from the infra-umbilical port site. Imaging of the lump revealed an intra-abdominal fluid collection communicating with the umbilicus. A diagnostic laparoscopy uncovered significant pus in the peritoneal cavity and a gallbladder remnant with multiple gallstones. A combination of sharp and blunt dissections was used to free the gallbladder remnant from its adherent surroundings for removal. A peritoneal washout was performed following extraction of the retained gallstones.

The patient’s presentation could be traced back to her laparoscopic cholecystectomy where it was confirmed that the retrieval bag ruptured during removal. This would have transected the gallbladder, causing its remnants and associated gallstones to be retained in the peritoneal cavity.

**Discussion:**

Retrieval bag rupture can result in retained gallbladder remnants in the peritoneal cavity. Abdominal abscess can manifest months after the initial operation.

**Conclusion:**

Retrieval bags should be inspected following removal to ensure it is completely intact. Surgeons should consider extending the fascial incision if there is any difficulty during removal. Any damage to the retrieval bag mandates immediate pneumoperitoneum for further exploration of retained products. Governance bodies should incorporate practice guidelines related to retrieval bag rupture as these are currently not present.

## Highlights

• Retrieval bag rupture during gallbladder removal is rare and its complications has never been reported.
• Retained gallbladder remnants and gallstones can occur secondary to retrieval bag rupture.
• This can result in intra-abdominal abscesses that can manifest months after the initial operation.
• Surgeons should always inspect the retrieval bag after removal to ensure it is completely intact.
• Any damage to the retrieval bag mandates immediate pneumoperitoneum for exploration of retained products.

## 1 Introduction

Retrieval bags are commonly used during laparoscopic cholecystectomies to reduce the risk of gallstone spillage and malignant seeding during removal of the gallbladder from the peritoneal cavity [1,2]. Since their introduction, there have been a variety of different retrieval bags described in the literature. These range from self-made gloves to several types of commercially manufactured bags [[3], [4], [5]]. Despite their common use in laparoscopic cholecystectomies, retrieval bags can sometimes complicate gallbladder removal. Injuries to abdominopelvic organs have been reported with retrieval bags in other laparoscopic surgeries such as nephrectomies [6]. Extension of the fascial incision appear more prevalent when laparoscopic bags are used, and this increases the risk of port site infections and hernias [7].

Nonetheless, most surgeons still favour the use of retrieval bags during laparoscopic cholecystectomies. Complications related to gallstone spillage and port site metastases are well documented in the literature and can be prevented through a retrieval bag [8]. Failure to clear the abdominal cavity from gallstones and bile may result in the development of wound infection, abscess, and peritonitis [9,10]. Retrieval bags are a cost-effective method to prevent these complications and most possess a short learning curve [11]. Rarely, the use retrieval bags may result in bile and gallstone spillage themselves. This can occur if the bag were to rupture under extreme tensile force during extraction through a small port site that has not been extended to accommodate the extra width. The risk of retrieval bag rupture is rare but has been reported to be as high as 1.4% [7]. Complications related to retrieval bag rupture have never been documented but can arguably be more serious than simple bile or gallstone spillage as they can also result in retained gallbladder remnants. We herein discuss a case where a gallbladder remnant was left in the abdominal cavity following rupture of a retrieval bag that occurred during laparoscopic cholecystectomy. The complications arising from this are described in this paper. This work has been reported in line with the SCARE criteria [12].

## 2 Case presentation

A 17-year-old female presented with a tender periumbilical mass three months post-laparoscopic cholecystectomy for symptomatic cholelithiasis. The mass was noticed two days prior to her presentation and appeared just deep to the infra-umbilical incision site utilised in her laparoscopic cholecystectomy. It measured 4 × 2 cm and was extremely tender and warm on palpation. The patient stated she was lethargic but denied any other symptoms. Her vitals were stable on admission (Temp 36.3 °C; RR 20; SpO_2_ 100% RA; HR 62; BP 122/78) and she appeared systemically well. Biochemically, she had a raised white cell count (17.7) and C-reactive protein (65).

**Fig. 1:**
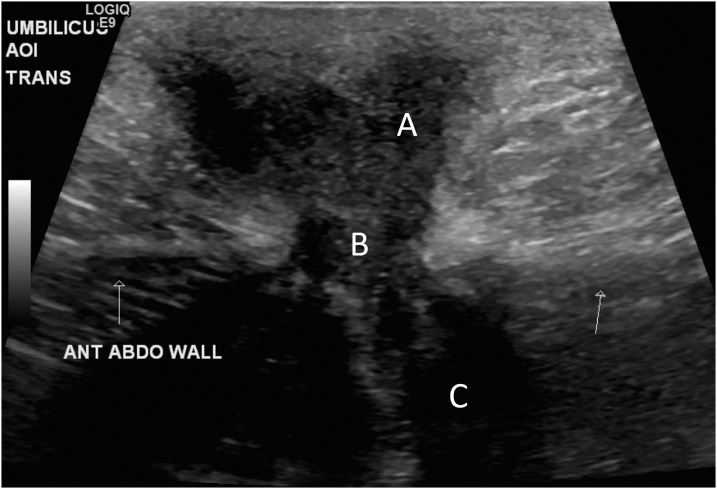
Ultrasound demonstrating hypoechoic fluid collection in the anterior abdominal wall (labelled A), communicating via a defect (labelled B), with an underlying hyperechoic structure (labelled C).

**Fig. 2:**
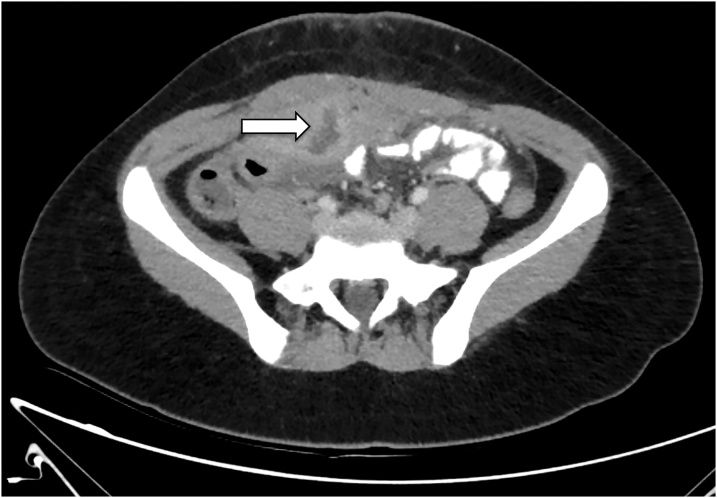
Intra-abdominal fluid collection seen as irregular hypodense lesion with peripheral enhancement.

Focused ultrasonography of the region demonstrated a heterogeneous predominantly hypoechoic fluid collection in the anterior abdominal wall just below the infra-umbilical incision site. This appears to communicate with a hyperechoic structure just below the abdominal wall (Fig. 1). Discussion with the radiologist suggested that these findings may be due to post-surgical soft tissue changes or an incisional hernia, but these differentials were considered unlikely in the context of the patient’s clinical presentation. An abdominal and pelvic CT was performed for further clarification and this revealed an intra-abdominal collection draining into the umbilicus (Fig. 2).

The patient returned to theatres for exploration of her umbilical wound. A re-incision of her previous infra-umbilical port site resulted in direct contact with an abscess lying immediately deep to it. Significant pus and gallstones were noted in the cavity along with a piece of tissue that was later histopathologically confirmed to be a gallbladder remnant. The cavity was removed of all its content and washed with hydrogen peroxide and normal saline. Inspection of the underlying fascia was shown to be intact, so the intraperitoneal contents were not explored. The wound was left opened post-operatively and packed with saline soaked gauzes daily for delayed primary closure. On discharge, the wound appeared uninflamed and the patient was instructed to have daily dressing changes in the community.

**Fig. 3:**
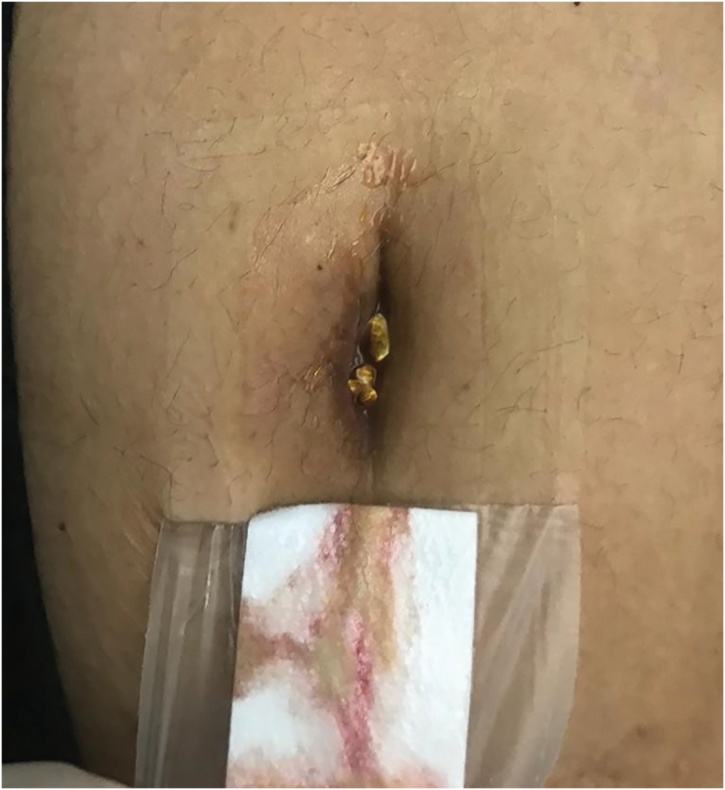
Gallstones expressing from the patient’s umbilical wound.

**Fig. 4:**
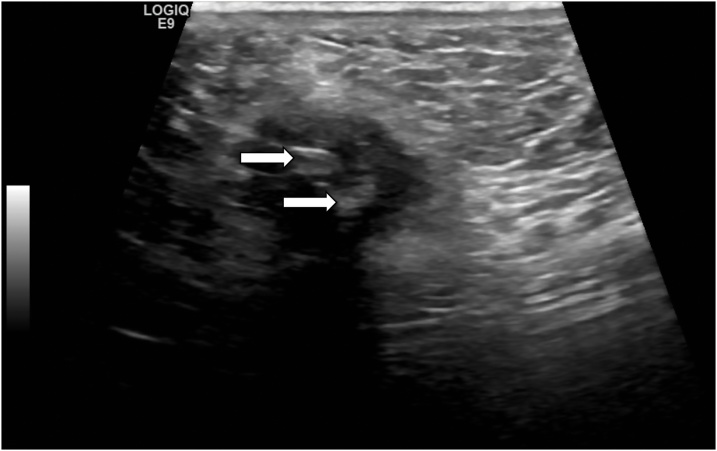
Ultrasound showing two echogenic masses representing gallstones.

**Fig. 5:**
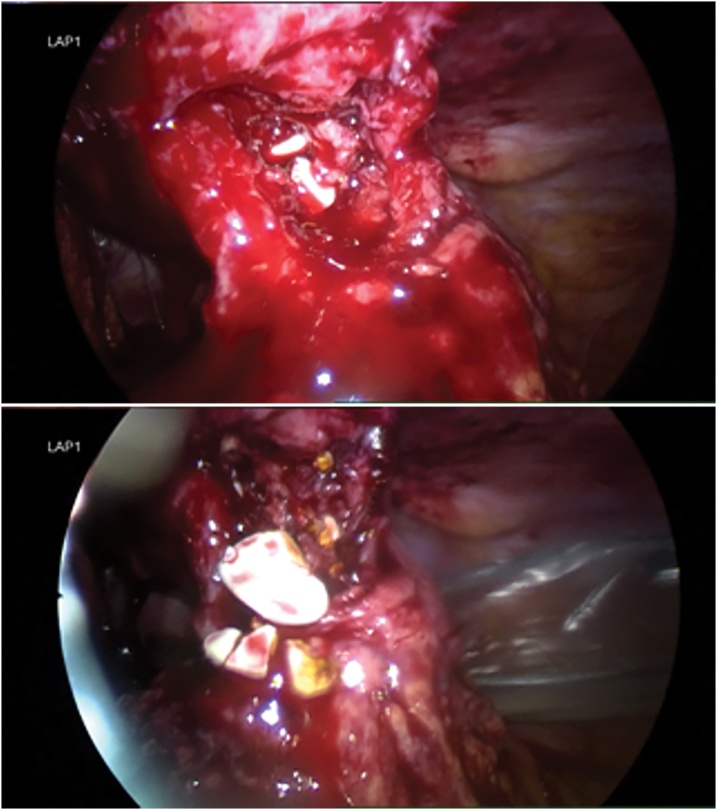
Intraoperative images showing retained gallbladder in the peritoneal cavity with associated gallstones.

Unfortunately, the patient re-presented to hospital 5 days following discharge. She complained about gallstones being expressed from her poorly healed wound (Fig. 3). A repeat ultrasonography of the abdominal wall revealed the presence of two echogenic masses representing gallstones (Fig. 4). The patient underwent an immediate diagnostic laparoscopy, entering the peritoneal cavity via her previous incisions utilised in her laparoscopic cholecystectomy. This revealed a retained gallbladder with numerous cholesterol stones (Fig. 5). A combination of sharp and blunt dissection delineated the retained gallbladder from adherent omentum and small bowel loops. All retained tissue and stones were collected and successfully removed. Postoperatively, the patient completed an one week course of amoxicillin/clavulanic acid 875/125 mg twice daily and made an uneventful recovery with delayed primary closure of her umbilicus.

The root cause of this patient’s presentation was traced back to her laparoscopic cholecystectomy performed three months prior to her initial presentation. The patient’s operative report noted difficulty removing the gallbladder retrieval bag from the infra-umbilical incision site but did not explicitly state any complications. On discussion with the primary and assisting surgeons who performed the laparoscopic cholecystectomy, it was clear that the retrieval bag used to remove the gallbladder from the peritoneal cavity ruptured as it was pulled from the infra-umbilical port site. This would have transected the gallbladder, causing its remnants and associated gallstones to be retained in the peritoneal cavity.

## 3 Discussion

Complications related to retrieval bag rupture are exceedingly rare and have never been previously reported in the literature. Our case demonstrates that gallbladder remnants and gallstones can be retained in the abdominal cavity following retrieval bag rupture. Abdominal abscess and port site infection secondary to these retained products can manifest months after the initial operation. It is worth noting that the retained gallbladder in our case was adherent to the surrounding omentum and small bowel, predisposing the patient to a potential obstruction or volvulus.

The remnant gallbladder that was found in our patient’s abdominal cavity was particularly unique because it was not a result of a partial cholecystectomy. Retained gallbladders secondary to partial cholecystectomies have been previously reported and are always found attached to the bile duct, which was not the case with our patient [13]. Reasons for a partial cholecystectomy include “difficult” gallbladders, surrounding adhesions, or confounding anatomy such as congenital duplication [14,15]. Retained gallbladders from partial cholecystectomies may result in abdominal abscess formation, but these tend to occur in the subhepatic region and not the periumbilical area as seen in our patient [16].

The use of retrieval bags in elective laparoscopic cholecystectomy have been viewed by some as unnecessary [7]. Bile and gallstone spillage during extraction do not occur nearly as frequently as during the operation itself, when iatrogenic gallbladder perforation can take place [17]. Complications from bile and gallstone spillage are rare according to large studies looking at overall outcomes of laparoscopic cholecystectomies [[17], [18], [19]]. Regina and colleagues [20] recently performed a meta-analysis, which demonstrated that retrieval bags do not reduce the rate of infection during gallbladder extraction. However, their paper excluded cases of cholecystitis and suspected carcinoma, presenting a biased perspective against retrieval bags. Until further research warrants otherwise, extraction of the gallbladder should be ideally performed with a retrieval bag.

## 4 Conclusion

Our case highlights the need to standardize practice related to the use of retrieval bags during extraction of the gallbladder. Although retrieval bag rupture is rare, regulatory bodies should consider incorporating practice guidelines to prevent associated complications. We strongly encourage reporting of other complications related to retrieval bag rupture to highlight the importance of ensuring the bag remains intact following removal from the peritoneal cavity. Any damage to the retrieval bag mandates immediate pneumoperitoneum for further exploration of retained products. To reduce the risk of retrieval bag rupture, surgeons should consider extending the fascial incision if there is any difficulty during removal. In our case, the original surgeon removed the retrieval bag via a 5 mm port site. While extending the fascial incision may increase the risk of post-operative pain and port site hernia, these risks are insignificant compared to the complications that can arise from a ruptured retrieval bag.

## Conflicts of interest

The authors have no conflicts of interest to declare.

## Funding

This case report did not receive any funding.

## Ethical approval

This is a case report and is exempted from ethical approval at our institution.

## Consent

Written informed consent was obtained from the patient for publication of this case report and accompanying images. A copy of the written consent is available for review by the Editor-in-Chief of this journal on request.

## Author contribution

Dr Roy Huynh: Care of patient described in case report, conception and design of study, acquisition of data, analysis and interpretation of data, drafting the manuscript, revising the manuscript for important intellectual content, approval of final manuscript.

Dr Mark Magdy: Care of patient described in case report, conception and design of study, acquisition of data, analysis and interpretation of data, drafting the manuscript, revising the manuscript for important intellectual content, approval of final manuscript.

Dr Lucia Saliba: Analysis and interpretation of data, revising the manuscript for important intellectual content, care of patient described in case report, advice and council on case report, approval of final manuscript.

Dr Ken Loi: Acquisition of data, analysis and interpretation of data, supervision of case report, revising the manuscript for important intellectual content, supervision of case report, approval of final manuscript.

## Registration of research studies

Not applicable.

## Guarantor

Dr Roy Huynh.

## Provenance and peer review

Not commissioned, externally peer-reviewed.
